# Supplementary material for: Prognosis and Treatment Outcomes of Bone Metastasis in Gallbladder Adenocarcinoma: A SEER-Based Study
Source: Cancers (Basel). 2023 Oct 19;15(20):5055. doi: 10.3390/cancers15205055 (PMC10605374; doi:10.3390/cancers15205055)
Supplement: Supplementary file 1 [file cancers-15-05055-s001.zip › cancers-2653096-supplementary.pdf]

SUPPLEMENTARY FILES

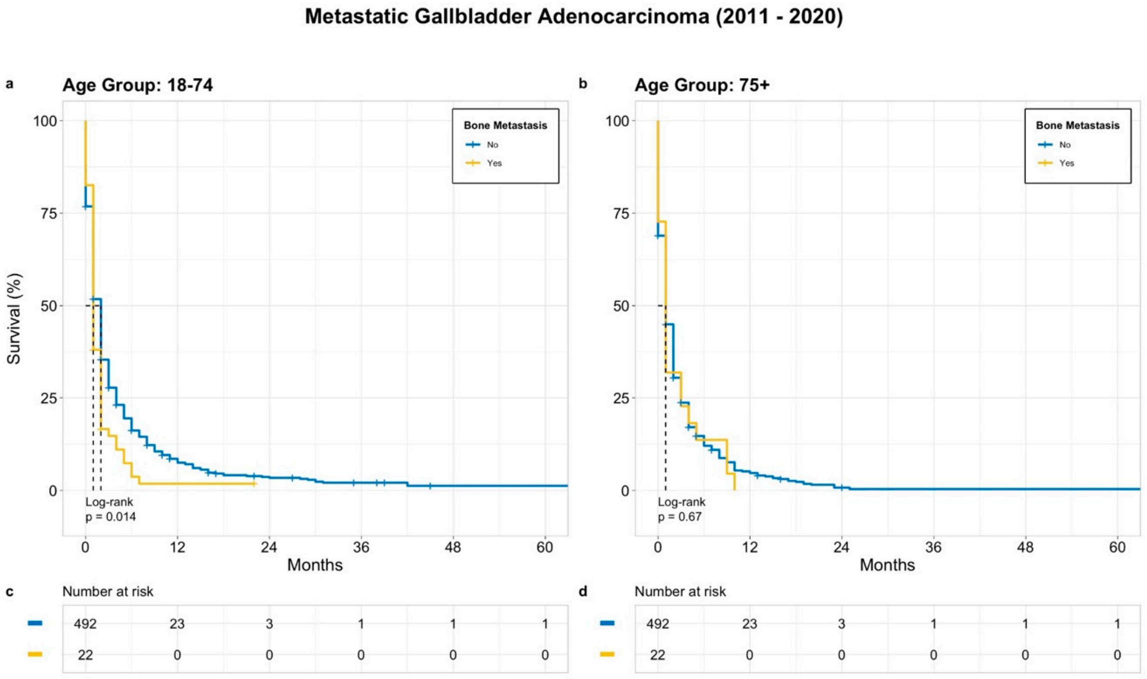

Figure S1. OS in patients with untreated metastatic GB adenocarcinoma based on bone metastasis; (a) age 18-74 years, (b) age 75+ years, (c,d) denotes patients at risk at a given time point.

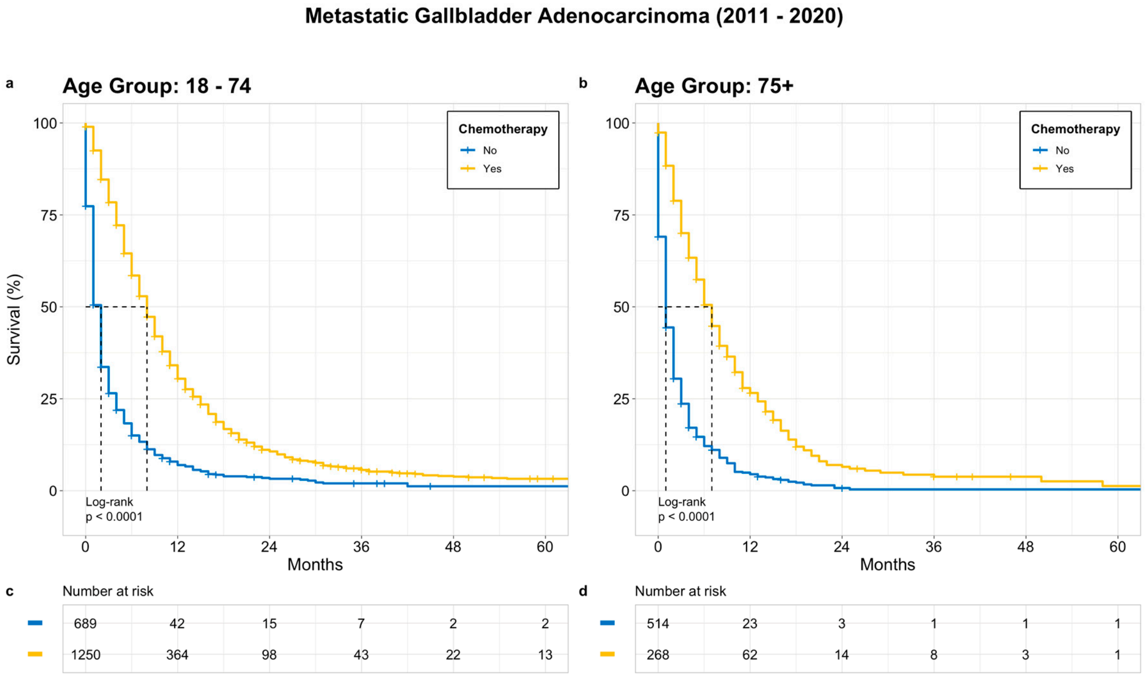

Figure S2. OS in patients with metastatic GBC based on receipt of chemotherapy; (a) age 18-74 years, (b) age 75+ years, (c,d) denotes patients at risk at a given time point.
